# The Impact of Previsit Contextual Data Collection on Patient-Provider Communication and Patient Activation: Study Protocol for a Randomized Controlled Trial

**DOI:** 10.2196/20309

**Published:** 2020-09-23

**Authors:** Jeana M Holt, Rachel Cusatis, Aaron Winn, Onur Asan, Charles Spanbauer, Joni S Williams, Kathryn E Flynn, Melek Somai, Purushottam Laud, Bradley H Crotty

**Affiliations:** 1 College of Nursing University of Wisconsin-Milwaukee Milwaukee, WI United States; 2 Department of Family & Community Medicine Medical College of Wisconsin Milwaukee, WI United States; 3 Hematology and Oncology Department of Medicine Medical College of Wisconsin Milwaukee, WI United States; 4 School of Pharmacy Medical College of Wisconsin Milwaukee, WI United States; 5 School of Systems and Enterprises Stevens Institute of Technology Hoboken, NJ United States; 6 Institute for Health and Equity Medical College of Wisconsin Milwaukee, WI United States; 7 Department of Medicine Medical College of Wisconsin Center for Advancing Population Science Milwaukee, WI United States; 8 Department of Medicine Medical College of Wisconsin Milwaukee, WI United States

**Keywords:** physician-patient relations, consumer health informatics, patient participation, vulnerable populations, randomized controlled trial, patient-centered care, mobile phone

## Abstract

**Background:**

Patient-centered care is respectful of and responsive to individual patient preferences, needs, and values. To provide patient-centered care, clinicians need to know and incorporate patients’ context into their communication and care with patients. Patient contextual data (PCD) encompass social determinants of health and patients’ needs, values, goals, and preferences relevant to their care. PCD can be challenging to collect as a routine component of the time-limited primary care visit.

**Objective:**

This study aims to determine if patient-provider communication and patient activation are different for patient users and patient nonusers of an electronic health record (EHR)–integrated PCD tool and assess if the impact of using PCD on patient-provider communication and patient activation differs for Black and White patients.

**Methods:**

We describe a randomized controlled trial of a prospective cohort of non-Hispanic White and Black patients who receive primary care services at a midwestern academic health care system in the United States. We will evaluate whether providing PCD through a consumer informatics tool enhances patient-provider communication, as measured by the Communication Assessment Tool, and we will evaluate patient activation, as measured by the Patient Activation Measure for PCD tool users and nonusers. Furthermore, owing to racial disparities in care and communication, we seek to determine if the adoption and use of the tool might narrow the differences between patient groups.

**Results:**

The trial was funded in November 2017 and received local ethics review approval in February 2019. The study began recruitment in April 2019 and enrollment concluded in October 2019 with 301 participants. The analysis was completed in May 2020, and trial results are expected to be published in winter 2020.

**Conclusions:**

Recently, there has been increased attention to the role of health information technology tools to enable patients to collaborate with providers through the sharing of PCD. The adoption of such tools may overcome the barriers of current EHRs by directly engaging patients to submit their contextual data. Effectively, these tools would support the EHR in providing a more holistic understanding of the patient. Research further supports that individuals who have robust digital engagement using consumer informatics tools have higher participation in treatment follow-up and self-care across populations. Therefore, it is critical to investigate interventions that elicit and share patients’ social risks and care preferences with the health care team as a mechanism to improve individualized care and reduce the gap in health outcomes.

**Trial Registration:**

ClinicalTrials.gov NCT03766841; https://clinicaltrials.gov/ct2/show/NCT03766841

**International Registered Report Identifier (IRRID):**

RR1-10.2196/20309

## Introduction

### Background

Over the past few decades, health care has been shifting from a paternalistic to patient-centered model that values patient engagement and shared decision making [1,2]. These values align with the patient-centered care model, where clinicians provide care that is tailored to the distinct needs of the patient. It is based on the development of respectful and dignified therapeutic relationships [3].

To provide patient-centered care, clinicians need to know and incorporate the patients’ context into their communication and care with patients. Patient contextual data (PCD) encompass social determinants of health (SDH) [4] and further comprise patients’ needs, values, goals, and preferences relevant to their care [5]. In the primary care setting, clinicians address most patients’ health care needs through a sustained partnership with patients and within the context of family and community [6]. Therefore, care teams must have access to data about the patients’ perspectives, values, and other contextual considerations to tailor patient-centered conversations and clinical decisions [1,6,7]. PCD can facilitate team-based care by enabling health care team members to build a rapport quickly and to connect with patients on a humanistic level [8].

Evidence suggests that connecting with patients can bolster patient activation. In a cross-sectional study, individuals at the highest level of activation (level 4) received relevant preventative cancer screenings, had 5 of 6 clinical indicators in the normal range, and did not engage in unhealthy behaviors (tobacco smoking and obesity) at statistically significantly higher rates compared with individuals in the lowest level of activation (level 1) [9]. A longitudinal study affirmed the results that indicated that people at the highest level of activation had significantly higher odds of guideline-concordant high-density lipoproteins and serum triglyceride levels, normal Patient Health Questionnaire-9 scores, not smoking, not being obese, having no Emergency Department visits, and having no hospitalizations in the 2-year follow-up period compared with people at the lowest levels of activation [9,10]. These outcomes translated to lower health care costs, with a projected 31% decrease in costs for people who were most activated (level 4) than those who were least activated (levels 1 and 2) [10].

Despite evidence indicating that connecting with patients improves patient outcomes and reduces health care costs [9,10], PCD are often not collected as a routine component of care [11]. A barrier to the integration of PCD is linked to the current limitation of electronic health record (EHR) systems in integrating and facilitating the retrieval of social risks and care preference data [12], even if collected as unstructured data within clinical notes. Clinicians face several limitations in terms of time [5,13] allocated to clinical visits and tools to gather a comprehensive understanding of their patients’ needs, values, preferences, goals, and concerns. System-level barriers lead to missed opportunities to individualize care and act upon PCD that might have a substantial impact on patient outcomes and the experience of care [14].

Previous research has demonstrated that patients reveal more sensitive information via health information technology than during patient visits [15]. Studies show that unvoiced concerns and goals for care disproportionately relate to the patients’ experience of illness [16,17], patients’ expectations of treatment [18], or psychosocial concerns [17,19-21]. These *contextual errors* (ie, disregard of PCD in care planning) [14] are more costly to the health care system than biomedical errors (ie, guideline-discordant care) [11]. Conversely, when providers incorporate PCD into the care context, patients’ engagement in their self-management and adherence to the agreed-upon care plan increases [22]. Furthermore, when the health care team collects and incorporates PCD during a visit, it facilitates rapport building and aligns patient and provider goals [5].

### Strategies to Mitigate Disparities

Research indicates that there are ethnic and racial differences in the adoption of consumer informatics tools [23-26]. In a study of a national sample of US adults, ethnic and racial minorities were less likely to be invited to use a patient portal than ethnic and racial majority populations [27]. Furthermore, individuals who did not use a patient portal were more likely to be unemployed, receive Medicaid insurance, have less than a college-level education, did not have a regular health care provider, were male, and aged 65 years or above [26]. As patient portal usage has been shown to be associated with improved quality measures and is thought to contribute positively to patient safety, digital tools should be assessed for their capability to be adopted by a wide range of the population and to narrow, rather than grow, the gaps in care across groups [28].

Given that disparities exist in the adoption and use of consumer informatics tools [27,29,30], researchers must evaluate ways to reach vulnerable populations when testing new consumer information technologies. Current trends suggest that internet access is no longer the main cause of the digital divide [31]. Instead, some patients lack the knowledge, skills, and confidence in using technology [32]. Providers may be key to reduce differences in consumer technology use by inviting all patients to use the new technology, discussing privacy and security concerns and providing resources tailored to a low health literacy level, on use [26]. Additional strategies to reach the most vulnerable individuals and across racial groups include developers employing patient-centered design strategies such as a simple, clean, and aesthetically appealing interface [33]; incorporating patient education on how to use the technology [34]; and promoting the new technology in various ways [35]. Conceivably, introducing a new consumer informatics technology designed to improve patient activation and communication may not achieve the desired rates of adoption, unless health care team members actively promote and assist in the use of the technology [8,25,35,36].

### Study Objectives

In this randomized controlled trial, we aim to evaluate the influence of PCD, collected using a consumer informatics tool, for previsit planning and routine clinical visit discussions with the health care team. The goal is to compare postvisit patient-provider communication and changes in patient activation among patient users and nonusers of the PCD tool, accounting for differences between non-Hispanic White and Black participants (hereafter White and Black). We will measure these constructs using 2 validated measures, the Communication Assessment Tool (CAT) [37] and the Patient Activation Measure (PAM) [38]. Furthermore, we will evaluate the impact by race by determining whether PCD could help mitigate any baseline differences in patient activation and postvisit patient-provider communication between White and Black patients.

We hypothesize that inviting patients directly to submit this information may help with several factors, including activation (patient ready to manage their health and care) and communication (helps prepare perspective and helps the clinician identify salient points).

The primary aims of this trial are to (1) assess the effects of using PCD on patient-provider communication (primary outcome) and patient activation (secondary outcome) and examine whether the effects are different for Black and White patients, accounting for age, gender, and other patient factors and (2) evaluate whether baseline measures of patient-provider communication and patient activation modify the effectiveness of PCD in improving either outcomes, accounting for age, gender, and other patient factors.

We have 2 outcomes of interest: (1) patient-provider communication measured using the CAT [37] and (2) patient activation measured using the PAM [38].

## Methods

### Study Design

This trial will assess the impact of incorporating PCD on patient-provider communication and patient activation of Black and White participants. The trial is registered at ClinicalTrials.gov (NCT03766841). The health network’s ethics review board approved this trial (registered project PRO00031177). The study protocol adheres to the Standard Protocol Items: Recommendations for Interventional Trials (SPIRIT) 2013 [39] checklist (Multimedia Appendix 1).

**Figure 1:**
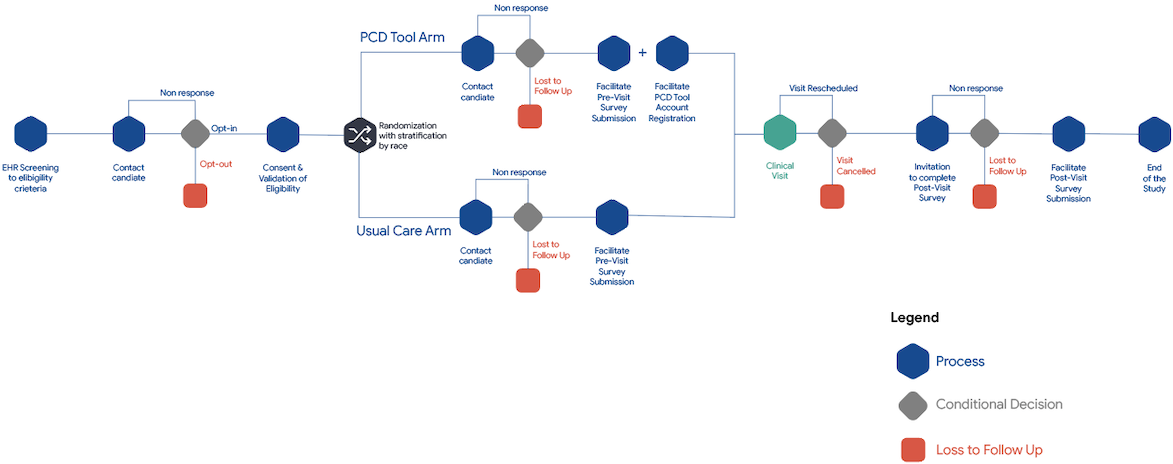
Randomized controlled trial study design.

Using an experimental study design, we will recruit a prospective cohort of eligible Black and White patients from primary care clinic sites randomized to intervention (invitation to use the PCD tool with facilitated enrollment) or usual care (invitation to use the PCD tool only) and administer questionnaires at baseline and after their primary care visit. The questionnaires assess the perceptions of visit communication and patient activation. The survey results will be adjusted for previsit measures of communication as the CAT [37] is only validated as a postvisit measure of patient-provider communication. Figure 1 presents the randomized controlled trial study design.

### Sample Size Determination and Randomization

An a priori power analysis was performed to estimate the required sample size using G*Power 3 [40,41] based on the study’s primary outcome, the CAT [37]. These were conducted for the more straightforward two-sample *t* test procedure, as this is known to yield a conservative assessment of power. For a two-sided test at α=.05, a total sample size of 200 results in 80% power for a standardized effect size of 0.4 and 94% power for an effect size of 0.5. Increasing the sample size to 250 raises the power to 88% and 98%, respectively. A sample size of 250 provides 79% power to detect a standardized effect size of 0.35. To account for up to 20% potential dropout over time, we aim to enroll 300 participants (targeting 150 Black and 150 White participants). Once participants provide consent, REDCap (Research Electronic Data Capture) [40] will randomize them into 1 of 2 experimental arms: (1) PCD tool (ie, facilitated enrollment for PCD tool intervention) or (2) usual care (ie, email invitation only for PCD tool). We will use stratified random sampling to ensure equal representation of Black and White participants in each arm. Stratified randomization prevents an imbalance of racial representation between arms.

### Randomization of Study Participants

#### Allocation Process

An allocation table was created using R to develop a block randomization scheme to balance arms and stratification by race. The block randomization scheme was then incorporated into the REDCap [40] system. Randomization is stratified by race in a 1:1 ratio, ensuring that we oversample Black participants based on population demographics. Blinding does not occur for either the participant or the study team. Participants are invited to join a communication study but are not told whether the study will focus on their use of the PCD tool.

#### Study Population

A total of 300 adults (≥18 years) with established primary care providers (ie, at least one visit in the previous 12 months with the same provider) from 2 academic and community-based primary care clinics of the Medical College of Wisconsin in Milwaukee, Wisconsin, United States, will be recruited for the study.

#### Inclusion Criteria

Eligible participants are individuals (1) aged 18 years or above, (2) who self-identify as non-Hispanic White or Black, (3) who speak and understand English, (4) who are willing and able to give informed consent, and (5) who at the time of the study enrollment period have an upcoming visit (1-4 weeks away), (6) whose appointment at one of the academic medical center’s primary care clinics, and (7) whose appointment is with an established provider (at least one previous appointment with the same provider within the last 12 months).

#### Recruitment

We will use consecutive convenience sampling to select every person who meets the inclusion criteria based on weekly EHR data reports. This sampling procedure minimizes selection bias (ie, volunteerism) [42]. Using the institution’s local informatics tools [43], we estimated the number of unique patients seen at the eligible clinics in 2016 to be 5200 Black and 13,750 non-Hispanic White patients. Restricting to unique patients with a preventive service encounter in 2016 (a conservative estimate as it excludes patients for whom a preventive exam was not billed, which includes most Medicare patients), there remain over 980 Black and 3725 non-Hispanic White patients who are eligible to participate.

We will contact the participants through a mailed letter or email. The invitation to participate describes the study as “a study to better understand and improve patients’ experiences of care and communication with their doctors.” Research staff will contact eligible participants by phone up to three times to answer questions, encourage participation, and facilitate the completion of the baseline survey. Recruitment will continue until we reach our target sample size of 300. We will collect the survey data using the REDCap system [44] hosted at the academic medical center.

#### Informed Consent

The informational letter participants receive as part of the informed consent process can be found in Multimedia Appendix 2.

#### Intervention

##### EHR-integrated PCD Tool

The Froedtert and Medical College of Wisconsin health network partnered with a digital health company, PatientWisdom Inc, to develop a digital web-based platform to engage with patients ahead of visits. After creating an account on the platform, each participant would be able to provide information about themselves and their situations (ie, PCD) as well as their agenda for the next visit through a mobile and web interface.

The PCD tool is a web-based application running on a Health Insurance Portability and Accountability Act–compliant platform. It has a responsive design that allows for ease of use across a range of devices, from desktops to tablets and smartphones. The tool was codeveloped by the health network, its patients, its clinicians, and an PatientWisdom, Inc. The consumer informatics tool draws upon deep experience and evidence in patient communication [37,45]. The tool invites patients to share *stories* about themselves, their health, and their care. For example, in the *My Self Story* section, patients share what they want their health care team to know about them as individuals, what brings them joy, and about the pressures in their life such as social and personal determinants of health. This section also includes the patient’s health-related priorities and goals and the barriers they experience in achieving them. In addition, in the *My Health Story* section, patients share questions or concerns they want to discuss with the care team, rate their health and provide reasons for the rating, and provide a perspective on how identified health issues affect their lives.

Furthermore, patients identify their preferences toward shared decision making and identify people who support them with health care decisions. Patients can access the application directly through a web address or through a drop-down menu embedded in the patient portal that provides a single sign-on experience for the patient. The latter process makes a direct linkage between the patient and the patient in the EHR. If the patient does not use the patient portal, a statistical matching algorithm links the accounts between the PCD tool and the EHR. After the linkage occurs, clinicians can click on an activity tab within the EHR to view a one-screen summary of the patient’s responses. From preliminary data, average engagement with the tool by patient PCD tool users is approximately 7 min per encounter.

##### EHR Integration

**Figure 2:**
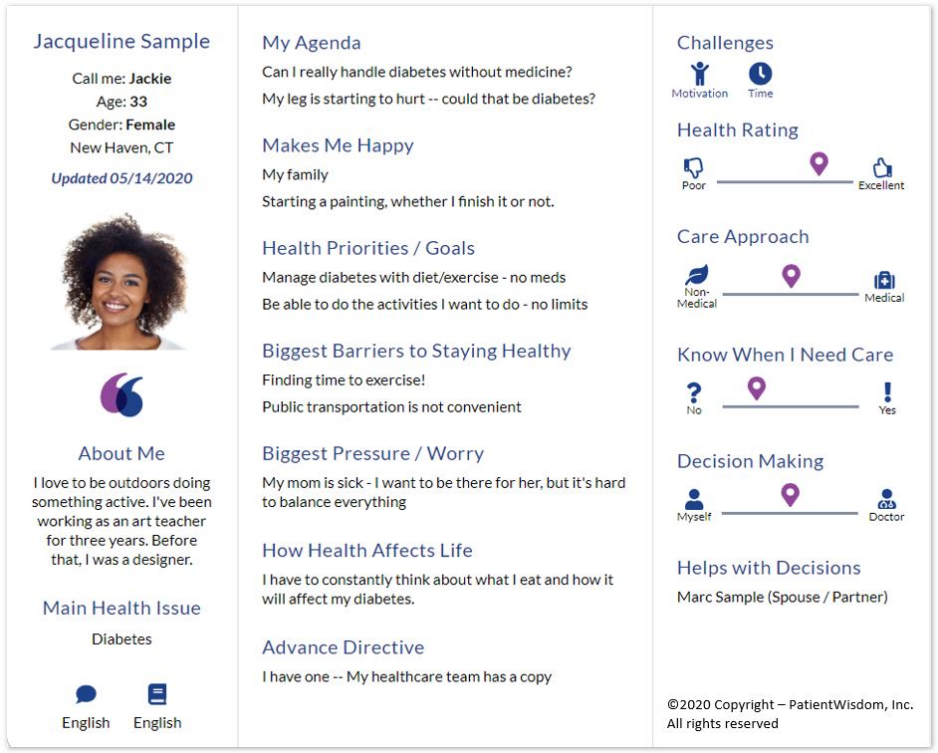
Patient contextual data tool one-screen summary.

The EHR-integrated PCD tool synthesizes information from the *My Self* and *My Health* stories to create an at-a-glance one-screen view (Figure 2; PCD tool one-screen summary) of the patient, their context, and what is relevant to them. The one-screen summary includes content to facilitate a personal connection and to efficiently grasp goals of care, agenda items, barriers, SDH, styles, and preferences. The 1-page summary highlights elements that the patient recently updated. From preliminary data, clinicians view the summary for approximately 1 min.

As the developers designed the tool to be asynchronous, they established an alert process to flag text and notify clinicians of critical patient data (eg, thoughts of suicide, domestic violence, or distressing symptoms) to guarantee timely interventions [5]. There are plans to transition the alert process to natural language processing once enough PCD are gathered for deep learning.

#### PCD Tool Arm

After completion of the previsit survey, all participants in the intervention group will receive an email with a link to the PCD tool for participants to complete their profile. Participants are given the option to complete their PCD profile independently or with assistance from one of the research staff. Providing the participant with options to complete their PCD profile ensures that the participant has access to the internet and a device. Facilitating the enrollment process may also overcome the current rates of adoption and use of the tool (4.7% in 2018) by being responsive to participants’ varying degrees of computer literacy and technical skills.

For participants who do not have an email address and decline to sign up for one, a paper survey will be used to collect the PCD and share them with the health care team at the time of the appointment. Although completion of a paper form loses some of the elements of the trial (PCD not integrated into the EHR), it decreases the chances of adding bias in the study as Blacks are less likely than Whites to have an email account [32,46].

##### Enrollment Process for PCD Tool Facilitation

The facilitation enrollment process includes a description of the PCD tool, followed by the study team member either assisting the participant in registering a PCD tool account using the link to the PCD tool site sent by email or describing how to register a PCD tool account through the patient’s portal. Next, the study team member will review the types of *stories* to share, in the domains of (1) information about me, (2) issues related to my care, (3) my upcoming visit agenda, and (4) barriers to care, to highlight all of them as important pieces of data. In addition, the study team member will share how to upload a picture to the profile. After completing the instructions, the study team member will share that the completed profile would then be available to the care team. The participant can then view how their profile would appear in their EHR via the one-screen summary.

The research team member will document the type of PCD tool facilitation (ie, email link only, over the telephone, or in person) for each participant and take field notes of each facilitation experience. Approximately 1 week before the primary care visit, the research staff will recontact the participant either by telephone or email up to three times. The research staff will thank the participant for being part of the study and inquire if they have questions regarding completing or updating their PCD tool profile. Research staff will also remind the participant to complete the profile, if it is not yet finished.

#### Usual Care Arm

Participants randomized to the usual care arm will complete their previsit survey, scheduled primary care visit, and postvisit survey. The only information regarding the PCD tool they receive before their visit is the email sent automatically by the EHR system to all patients at the academic medical center to create or update their PCD account 1 week before their appointment. For participants who did not have a previsit survey completed at least five days ahead of the appointment, a study team member will give a call to remind the participant to complete the survey as soon as possible. For participants who indicate a preference to complete the previsit survey over the telephone or in person, the study team member will read the survey items verbatim and complete the survey in REDCap.

For both arms, after the scheduled primary care clinic visit occurs, participants will receive up to 3 email reminders to complete the postvisit survey. A study team member will give a call to remind the participant to complete the postvisit survey if it is not completed after the third email reminder. For participants who indicate a preference to complete the postvisit survey over the telephone or in person, the study team member will read the survey items verbatim and complete the survey in REDCap.

#### Data Collection

We will use self-reported surveys to assess the differences in patient-provider communication and patient activation between the PCD tool and usual care arms and by race (Black and White). The primary outcome measure is patient-provider communication assessed using the CAT [37] after the visit. The 15-item measure is unidimensional and has high internal consistency (Cronbach α=.96), with readability at or below an eighth-grade level [37]. The psychometric properties of CAT were tested in a diverse sample. The testing revealed that the instrument has content and construct validity and reliably [37] measures patients’ perceptions of physicians’ interpersonal communication skills. We will examine individual items and the proportion of items with top ratings. The CAT was designed to be administered directly following a visit and is not yet validated in a retrospective context. Therefore, we will use the Clinician and Group Consumer Assessment of Healthcare Providers and Systems (CG-CAHPS) survey [47,48] communication composite questions validated for patients’ perceptions of communication with their provider within the past 12 months as the baseline communication measure. The outcome will be the CAT adjusted for the baseline CG-CAHPS score.

We will use the 13-item PAM [38] to assess changes in the secondary outcome and patient activation, examining the change in pre- and postvisit assessments. Conducted with a nationally representative sample, psychometric testing revealed that the 13-item PAM questionnaire yielded a strong Rasch person reliability score between .85 (real) and .87 (model), and the Cronbach α value was acceptable at .87 [38].

We will collect the following independent variables in the previsit survey: CG-CAHPS communication composite [47], Patient-Reported Outcomes Measurement Information System (PROMIS) Global Health [49], health literacy [50], technology use or technology acceptance [51], and sociodemographic characteristics. We will measure previsit patient-provider communication using the communication composite of the CG-CAHPS survey [48]. The CG-CAHPS communication composite has high internal consistency (Cronbach α=.89) [47], which was determined using a nationally representative sample of over 21,000 patients from 450 US practice sites. We will assess participants’ perceptions of their global health using the PROMIS 10-item Global Health Short Form, which includes scores on global physical health and global mental health. The PROMIS 10-item Global Health Short Form scales had internal consistency reliability coefficients of 0.81 for global physical health and 0.86 for global mental health in a large national survey [49]. Technology use or technology acceptance will be collected using the Health Information National Trends Survey 5, Cycle 1 [51]. Participants will report health literacy using a validated one-item tool [50]. Sociodemographic characteristics and other hypothesized predictors of the outcome measures include income, age, sex, gender identity, health insurance status, educational attainment, number and type of chronic conditions, and length of relationship with primary care provider (in months or years).

**Figure 3:**
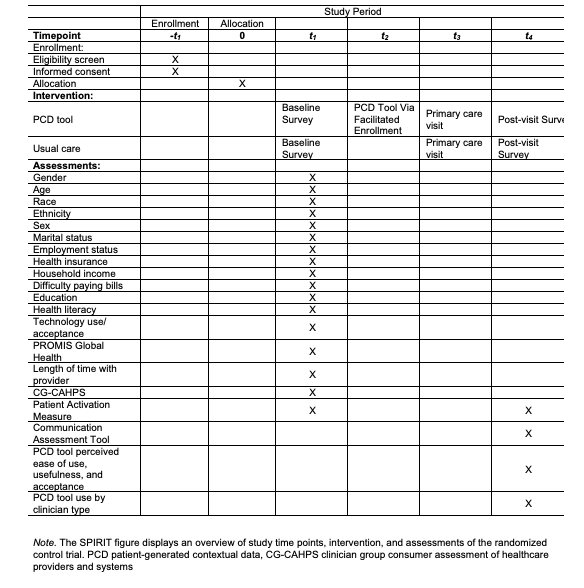
Randomized controlled trial Standard Protocol Items: Recommendations for Interventional Trials (SPIRIT).

We will monitor the use of the PCD tool in 3 ways. First, we will assess whether those in the PCD tool arm completed their profile before their appointment. Second, for participants in the usual care arm, we will determine if they created a profile after they entered the study, as all patients in the academic medical center have access to the PCD tool. Third, we will assess whether any of the care team members reviewed the participant’s PCD tool profile within one day before the appointment and on the day of the appointment. Figure 3 displays the Randomized Controlled Trial Standard Protocol Items: Recommendations for Interventional Trials (SPIRIT) [39] including an overview of study time points, intervention, and assessments of the randomized controlled trial.

#### Data Management

The research team will use REDCap for data management [44]. This system is a secure, web-based application designed by Vanderbilt University to support data collection for research. It provides data validation, audit trails, and automated export procedures to a variety of statistical packages. As necessary, branching logic and calculated fields will be created in the system to support data entry.

#### Data Monitoring

The data monitoring committee comprises the study team, the PCD tool’s implementation manager, and the Department of Medicine Safety Committee (DMSC). This pragmatic trial is of low risk, but several monitoring processes are in place to protect the participants. The participants are provided with the study team’s phone number and email address. Participants also have contact phone numbers of members of the ethics review board and can notify the principal investigator (PI) of any harm, which will be reported to the local institution. If a participant shares concerning data (eg, thoughts or actions of self-harm, domestic violence) in their PCD tool profile, the provider is alerted and contact with the participant is initiated. The study team will not conduct interim analyses because of the low-risk nature of the trial. There is an independent process for the DMSC to review all PCD tool data for quality in each quarter.

#### Ethical Considerations

The academic health network’s ethics review board approved the study before enrollment. The study PI will report changes to the protocol to ClinicalTrials.gov, local ethics review board, and all study team members. All study personnel have completed training on the protection of human subjects in research. Data will be stored on a secure server with physical, technical, and administrative access controls using the academic health system–approved REDCap software. Remote access is available over a secure network via encrypted connections to password-authorized users. The media will be kept in locked file cabinets in locked offices. Files with participant identifiers will be stripped of identifiers as soon as they are no longer needed. The study staff have no conflicts of interest. A subsidiary of the affiliated health system has an investment in the company that owns the PCD tool. However, the study staff are not directly employed by the health system nor have any financial ties to the company. There are no provisions for ancillary or postcare of the trial because of the nature of use of the PCD tool, which is currently available to the health system’s patients.

#### Analysis

The trial will evaluate the differences between the arms for change in PAM scores [38] and the CAT score (a postvisit measure) [37], adjusting for the CG-CAHPS score (a previsit measure). The research team will also assess differences in pre- and postvisit patient activation and postvisit patient-provider communication by race. Our primary analysis is an intention-to-treat (ITT) analysis, where every randomized participant is analyzed in the group to which they were randomly assigned [52,53]. Chi-square tests for categorical variables and independent sample *t* tests for continuous variables will be used to examine the differences between the groups at baseline and postvisit. Descriptive statistics (means and standard deviations) will be conducted for the following variables: age, CG-CAHPS communication composite score [47], and PROMIS 10-item Global Health Short Form [49]. Frequencies will be calculated for sex, gender identity, education, marital status, employment status, income, health insurance coverage and type, difficulty paying bills, health literacy, internet use, internet access, internet access location, and internet access device. We will also extract data from each participant’s EHR to calculate their Charlson Comorbidity Index [54] as a measure of morbidity. Previsit assessment of communication using CG-CAHPS [47] as a control variable and postvisit assessment of communication using the CAT [37] will be tested within and between groups using linear regression, controlling for covariates. A linear regression model will be used to determine the factors that predict changes in patient-provider communication and patient activation, controlling for covariates.

However, we expect that there will be some crossover and noncompliance between arms. For example, some individuals randomized to the PCD tool arm may not complete a profile, and some individuals not randomized to enroll in the PCD tool may create a profile. To overcome this limitation, we will conduct additional analyses that account for noncompliance and estimate the effect of the treatment-on-the-treated (TOT) instead of the ITT [55,56]. TOT is sometimes referred to as the local average treatment effect or as a *per-protocol* analysis. We will identify this using a two-stage least squares regression [57,58]. The first stage of the model estimates whether a person used the PCD tool during the follow-up period.

The second stage will identify the causal impact of using the PCD tool on outcomes (patient activation and patient-provider communication). To do this, we will model the predicted use of the PCD tool from stage 1 in the stage 2 model and use the results of this coefficient to interpret how the PCD tool impacts patient activation and patient-provider communication.

A missing value analysis will be conducted on the final data set to determine whether data were missing completely at random, missing at random, or missing not at random [59,60]. To reduce the likelihood of missing data biasing our results, we will use multiple imputation by chained equations to fill in missing data stratified by race [59]. The imputation algorithm will include participant demographics and clinical characteristics. Multiple imputation has been increasingly applied to clinical research to address the common problem of incomplete data sets [60].

For all aims, statistical analysis will be completed using SAS [61] procedures GLM and MIXED to assess and account for possible provider and center heterogeneity. A *P* value of <.05 is considered statistically significant.

#### Dissemination

We intend to write and publish 2 manuscripts (corresponding to each outcome, the CAT and the PAM), adhering to the International Committee of Medical Journal Editors [62] authorship recommendations. In addition, we will communicate the study results to the academic health system leadership, primary care clinics, developer of the tool, and the medical community.

#### Trial Status

The study on the impact of PCD on patient-provider communication and patient activation began recruitment on April 1, 2019. The trial ended recruitment on October 18, 2019.

## Results

The trial was funded in November 2017 and received local ethics review approval in February 2019. The study began recruitment in April 2019 and enrollment concluded in October 2019 with 301 participants. Analysis was completed in May 2020, and trial results are expected to be published in winter 2020.

## Discussion

### Role of Consumer Informatics

There is increasing attention on the role of health information technology and digital health tools to enable patients to collaborate with providers by sharing and acting upon PCD [12,63-70]. Adoption of consumer-facing informatics tools may overcome the barriers of current EHRs by directly engaging patients to share PCD. Moreover, with the advent of application programming interfaces and the increasing level of interoperability of EHR systems, these consumer applications can integrate PCD information into current EHR systems to make the data available for use by clinicians and health care teams [1,71]. In particular, consumer informatics tools that gather and share PCD are hypothesized to improve communication [21] and health outcomes [72,73]. Effectively, these tools would support the EHR in providing a more holistic understanding of the patient.

In an earlier study [8], digitally engaged patients reported that the completion of their profiles in a consumer informatics tool (ie PatientWisdom, Inc.) promoted reflection of their health goals, challenges, and priorities. The reflection led to actions toward goal attainment and targeted conversations with their health care team about issues important to them [8]. Research further supports that individuals who have robust digital engagement using consumer informatics tools have higher participation in treatment follow-up and self-care [74,75]. When care goals were aligned, racial and ethnic minority populations experienced improvements in patient-provider communication and decision quality outcomes similar to racial and ethnic majority populations [25,76]. Therefore, it is critical to understand whether interventions that elicit and share patients’ social risks and care preferences with the health care team serve as a mechanism to improve individualized care and lessen the gap in health outcomes.

### Summary, Strengths, Limitations, Contingency Strategies, and Alternative Designs

The clinical trial will provide crucial empirical evidence on the effects of a consumer informatics tool that elicits and aggregates PCD for use in the clinical exchange of patient-provider communication and patient activation across populations. The study will occur within the most racially segregated metropolitan area in the United States, where racial disparities in health and health care represent a significant public health concern [77-80]. The study sample may not reflect the population or the complex contextual issues associated with the area.

We acknowledge the following limitations and significant threats to the study and present contingency strategies. This research study will occur within 1 academic medical center, which may limit the generalizability of the results. To mitigate this limitation, we will recruit participants from various academic and community primary care clinics with different staff, providers, milieu, and the composition of patients who receive care. The participants in this study will be Black and White and limited to individuals who can speak English. This inclusion criterion excludes other diverse populations. This limitation is because of the population of patients who are served at the academic medical center. To reach equal racial representation of participants, we will oversample Black primary care patients. Recruitment difficulties for participation may occur. We employ several recommended strategies to recruit Black populations into this trial but lack other strategies such as community involvement and informational sessions [81]. Furthermore, the study may be threatened by volunteer bias, where the participants’ characteristics or outcomes differ from those of nonparticipants [41]. An efficacious strategy to improve participant recruitment and retention is to compensate individuals for their time [41]. Participants will receive a modest financial incentive for participation in this research project. The incentive is US $25 for each survey completed. The study team has also allocated substantial time and resources for personalized telephonic or in-person contact during recruitment, retention, and follow-up procedures. Researchers have successfully used these strategies to recruit and retain historically disenfranchised populations in clinical trials [41].

We also considered the alternative design of an efficiency trial with its advantages of high internal validity [53]. Although there are methodological advantages to this design, the real-life variability of clinical practice precludes the strict adherence to a study protocol mandated in an efficiency trial. Therefore, we chose a pragmatic clinical trial with somewhat diminished internal validity but a high degree of external validity of the results, which is valued in implementation research [53].

### Study Design Innovations

Health care stakeholders, clinicians, and patients increasingly call for the evaluation of clinically relevant interventions that are tested in heterogeneous clinical settings with the inclusion of diverse study participants [56,82]. In this clinical trial, we will test an intervention (PCD tool) that is deployed across an academic health network. We focus on understanding the differences by use, adjusting for problems with bias and self-selection of users for the PCD tool. The study design intends to overcome self-selection bias by creating randomization to treatment using various facilitation processes to improve the usage of the tool beyond its baseline. In addition to the ITT analysis, typical in pragmatic trials [55], we will conduct a TOT analysis [57,58] to model estimates of whether a participant used the PCD tool in the follow-up period and then identify the causal impact of using the PCD tool on outcomes (patient activation and patient-provider communication). In this study, the TOT analysis will adjust for participants’ nonadherence to the group assignment, a common occurrence in pragmatic trials [56].

### Conclusions

When patients’ preferences and life circumstances drive health care decisions, their quality of involvement in their care improves [9,83-85]. PCD are essential information that, when known and incorporated, may promote the development of a person-centered plan for care [14]. Therefore, interventions that test these relationships must be explored to understand how to optimize individuals’ involvement in self-care. Researchers must also investigate whether the outcomes differ between Black and White patients who experience different social, political, and economic injustices that affect health [86].

## Appendix

Multimedia Appendix 1 Standard Protocol Items: Recommendations for Interventional Trials (SPIRIT) Checklist.

Multimedia Appendix 2 Randomized controlled trial informed consent.

## Abbreviations

CG-CAHPS: Clinician and Group Consumer Assessment of Healthcare Providers and Systems
EHR: electronic health record
HHS: US Department of Health and Human Services
HRSA: Health Resources and Services Administration
PAM: Patient Activation Measure
PCD: patient contextual data
SDH: social determinants of health
