# Supplementary material for: The Impact of Previsit Contextual Data Collection on Patient-Provider Communication and Patient Activation: Study Protocol for a Randomized Controlled Trial
Source: JMIR Res Protoc. 2020 Sep 23;9(9):e20309. doi: 10.2196/20309 (PMC7542405; doi:10.2196/20309)
Supplement: Multimedia Appendix 2 [file resprot_v9i9e20309_app2.docx]

Randomized Controlled Trial Informed Consent

*If you choose to participate in this research study, we will invite you to (1) complete a short survey (Communication Study Survey) before your next scheduled visit at a Froedtert & Medical College of Wisconsin primary care clinic, (2) have a brief exchange with a research team member by phone OR email (your preference) to learn about a new initiative at Froedtert & the Medical College of Wisconsin, and then (3) complete a 2nd survey after your next scheduled primary care visit. Participation would also entail allowing the research team to look at your electronic health record for research purposes related to this communication study. We hope to include 300 people, so your answers and participation are really important to us. You are free to skip any questions or discontinue your participation in this research study at any time. Although you will not get personal benefit from taking part in this research study, your responses may help us understand how to improve communication between patients and clinicians.*

*Note.* The following instructions are given to the participants as an informational letter as part of the informed consent process.
